# Thermodynamics and kinetics in antibody resistance of the 501Y.V2 SARS-CoV-2 variant†

**DOI:** 10.1039/d1ra04134g

**Published:** 2021-10-13

**Authors:** Son Tung Ngo, Trung Hai Nguyen, Duc-Hung Pham, Nguyen Thanh Tung, Pham Cam Nam

**Affiliations:** Laboratory of Theoretical and Computational Biophysics, Ton Duc Thang University Ho Chi Minh City Vietnam ngosontung@tdtu.edu.vn; Faculty of Applied Sciences, Ton Duc Thang University Ho Chi Minh City Vietnam; Division of Immunobiology, Cincinnati Children's Hospital Medical Center Cincinnati 45229 OH USA; Institute of Materials Science, Vietnam Academy of Science and Technology Hanoi Vietnam; Graduate University of Science and Technology, Vietnam Academy of Science and Technology Hanoi Vietnam; Department of Chemical Engineering, The University of Da Nang, University of Science and Technology Da Nang City Vietnam pcnam@dut.udn.vn

## Abstract

Understanding the thermodynamics and kinetics of the binding process of an antibody to the SARS-CoV-2 receptor-binding domain (RBD) of the spike protein is very important for the development of COVID-19 vaccines. In particular, it is essential to understand how the binding mechanism may change under the effects of RBD mutations. In this context, we have demonstrated that the South African variant (B1.351 or 501Y.V2) can resist the neutralizing antibody (NAb). Three substitutions in the RBD including K417N, E484K, and N501Y alter the free energy landscape, binding pose, binding free energy, binding kinetics, hydrogen bonding, nonbonded contacts, and unbinding pathway of RBD + NAb complexes. The low binding affinity of NAb to 501Y.V2 RBD confirms the antibody resistance of the South African variant. Moreover, the fragment of NAb + RBD can be used as an affordable model to investigate changes in the binding process between the mutated RBD and antibodies.

## Abstract 2

Increasing FEL minima of 501Y.V2 RBD + antibody in comparison with the WT RBD systems imply that the complex 501Y.V2 RBD + antibody is more unstable than the WT one.

## Graphical abstract

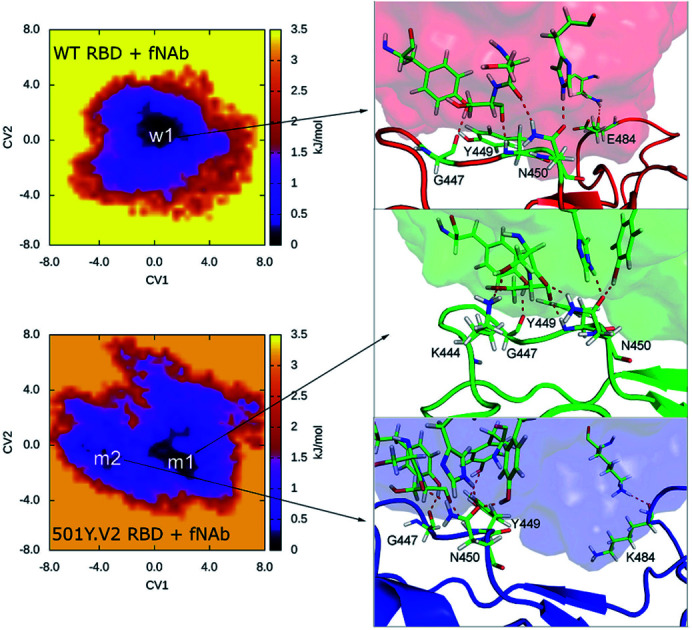

## Introduction

The novel β-coronavirus, SARS-CoV-2, whose sequence is similar to SARS-CoV-1 and MERS-CoV, which induced the human respiratory epidemic at the beginning of this century, is the cause of the human respiratory disease (COVID-19) pandemic worldwide.^1,2^ This virus has infected more than 160 million people and is associated with more than 3 million deaths.^3^ SARS-CoV-2 is a single-positive-strand RNA virus, whose genome encodes for four main components: spike, envelope, membrane, and nucleocapsid.^4,5^ The spike protein (S protein) of SARS-CoV-2 which is used by the virus to bind to human angiotensin-converting-enzyme 2 (ACE2), has been researched thoroughly. ACE2 is present in different tissues in the body, including the lung, heart and liver,^6^ and is employed by SARS-CoV-2 as a receptor to bind and infect human cells. The S trimer comprises three copies of S1 and S2 subunits. The S1 subunit contains 4 domains: S1A, S1B, S1C, and S1D, in which the S1B domain is also called the receptor-binding domain (RBD), which mediates the attachment of the spike protein to the target cell *via* binding to the ACE2 receptor.^7^ Once the RBD is in the ‘up’ conformation, it can recognize and bind to ACE2, which leads to conformational changes of the S2 subunit and enables SARS-CoV-2 to fuse with the cell membrane and to enter host cells.^1,7^

RBD is the main target of neutralizing antibodies (NAbs) which can be isolated from plasma of COVID-19 patients, immunoglobulin libraries, or immunized laboratory animal models.^1^ These NAbs can be roughly divided into four main classes, of which class 1s′ and class 2s′ RBD epitopes overlap with the ACE2-binding site, suggesting a neutralization mechanism that involves direct competition with ACE2. Class 1 antibodies, which are encoded by the immunoglobulin V-gene (VH3-53) segment with complementarity-determining regions 1 and 2 (CDRH1 and CDRH2) and a short CDRH3, are mostly elicited by SARS-CoV-2 infection. On the other hand, when class 2 antibodies also target site I^10,15^ which is also target epitopes of class 1 antibodies, they bind to RBD in both ‘up’ and down’ conformations of S protein.^1,8^ Additionally, class 3 antibodies bind outside ACE2 and recognize both up and down RBD, while class 4 antibodies comprise previously described antibodies that cannot block ACE2 and target only to RBD in ‘up’ conformation.^1^ Besides RBD, the N-terminal domain (NTD) of protein S is also a popular target for NAbs and many potent monoclonal antibodies directed against this region show great potential in clinical trials for COVID-19 treatment.^8^ The majority of these antibodies target a single immunodominant site on NTD, including the N1-loop (NTD N-terminus), N3-loop (supersite b-hairpin), and N5 loop (supersite loop). Subsets of these antibodies and NAbs in class 1 and class 3 form multi-donor classes, with a different set of VH germline restricted mode of spike recognition.^8^

Due to many reasons, including high transmissibility, the longevity of the pandemic, and encountering with immunocompromised hosts, SARS-CoV-2 undergoes different rounds of mutations, which has altered the structures of the virus, modulated its infectivity, and changed the antigenicity of the surface proteins.^9^ The variants, including United Kingdom (B1.1.7) and South African (B1.351 or 501Y.V2) variants have associated with increased transmissibility and possibly increased mortality.^8^ Especially, the SARS-CoV-2 lineage in South Africa, included nine mutations in the spike protein, seems to decrease the efficacy of NAb as well as Covid-19 vaccine efficacy of some vaccines currently being used.^10,11^ The mutations in B1.351 can be divided into two groups, one concentrates in NTD, including four substitutions and a deletion (L18F, D80A, D215G, Δ242–244, and R246I), and the other involves three substitutions in RBD (K417N, E484K, and N501Y).^12^ These changes induce S protein biological and structural alterations. Especially, mutation E484K is very critical, which can reduce the effect of NAb.^13^

**Fig. 1:**
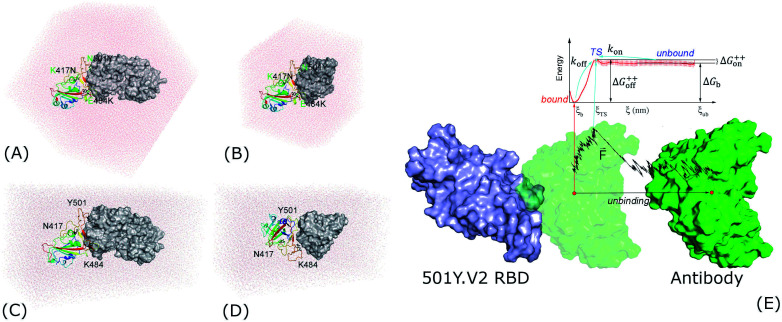
Starting structures of RBD + antibody systems. (A) 501Y.V2 RBD + NAb in MD simulations; (B) 501Y.V2 RBD + fNAb in MD simulations; (C) + (D) 501Y.V2 RBD + NAb/fNAb in biased MD simulations; (E) free energy scheme. The WT RBD + antibody complexes were formed an initial conformation similar to the 501Y.V2 one. The fNAb was mobilized from *bound* to *unbound* states *via* the fast pulling of ligand (FPL) calculations, then the free energy profile was calculated *via* US simulations.

Evaluating antibody resistance of the 501Y.V2 SARS-CoV-2 variant is greatly attractive to scientists.^8,10,11,14^ Understanding the physical insights into the process probably enhances the vaccine developments, but the knowledge is still limited. Therefore, in this context, atomistic simulations were carried out to reveal the insights at the atomic level of the binding process of NAb to 501Y.V2 SARS-CoV-2 RBD. For the first step, structural changes of the 501Y.V2 and wildtype (WT) SARS-CoV-2 RBD + NAb complexes were characterized *via* unbiased MD simulations. Thermodynamics and kinetics of the binding process were then revealed *via* biased MD simulations. Moreover, fragment of NAb (fNAb) is often used to study the binding of S protein/RBD to antibody.^15,16^ In this work we also investigated binding of fNAb (*cf.*Fig. 1E and S1 of the ESI file†) to RBD to evaluate the possibility of using fNAb in studying the influence of RBD mutations on the binding affinity instead of using NAb which is a larger molecule and costs more computing resources. Furthermore, it should be noted that glycosylation of RBD was neglected to clarify the interaction nature between RBD + antibodies, although glycans play an important role in the modulation of the spike conformational dynamics.^17–19^ Details of simulations were described in Fig. 1 and the ESI file.†

## Computational methods

### Structure of SARS-CoV-2 antibodies and RBD

The three-dimensional structure of SARS-CoV-2 RBD and their antibodies NAb was found from the Protein Data Bank (PDB) with the identities of 7BWJ.^20^ The fNAb was extracted from NAb as showed in Fig. 1E. The resolution of 7BWJ and 2.85 Å. Moreover, as mentioned above, it should be noted that the new variant of the SARS-CoV-2 in South Africa, B1.351 or 501Y.V2, forms eight changes in the spike protein. There are four substitutions and a deletion in the N-terminal domain (NTD) including L18F, D80A, D215G, Δ242–244, and R246I. Consequently, three substitutions were found in RBD involving K417N, E484K, and N501Y. The RBD structure with three substitutions was thus prepared *via* changing three residues of 7BWJ using the PyMOL mutagen tools.^21^ Besides, 50Y.V2 RBD with glycan was obtained from PDB ID 7LYQ,^22^ in which glycan linked with the residue Asn343.

### Molecular dynamics simulations

The atomistic simulation was performed using the GROMACS version 5.1.5 with general-purpose computing on graphics processing units.^23^ The protein, antibody, and neutralized ions were parameterized *via* the Amber99SB-iLDN force field^24^ since it is a suitable force field for free energy calculation.^25^ The TIP3P water model was chosen to simulate the water molecule.^26^ The system's configurations were shown and reported in detail in Fig. 1A and B and Table S1 of the ESI.†

The MD simulation parameters were taken from the previous works.^27,28^ However, in particular, the integration time step was taken to be 3 femtoseconds. A non-bonded pair interaction were cutoff at a radius of 0.9 nm, in which the electrostatic interaction was calculated using the fast Particle-Mesh Ewald electrostatics approach^29^ as well as the van der Waals (vdW) interaction was computed using the cut-off scheme. The solvated complex was initially optimized using the energy minimization *via* the steepest descent method. The minimized system was then relaxed in NVT and NPT ensembles with a length of 100 ps each simulation. During NVT and NPT simulations, the integral was attempted every 1 femtosecond. The equilibrium snapshots obtained *via* NPT simulations were used as starting conformations of MD simulations. The conventional MD simulations were performed with interval 100 ns and repeated 4 times independently. These independent trajectories thus have the same initial conformation but different generated velocities.

### Biased molecular dynamics simulations

#### Steered-MD (SMD) simulation

Representative structures of RBD + antibody systems, which were obtained *via* MD simulations, were employed as initial structures of FPL simulations. The complexes were reinserted into the rectangular PBC box for saving the computing resources. The configuration information was described in Fig. 1C, D and Table S1.† The FPL simulations were carried out to generate unbinding conformations of the systems, which were used as starting shapes of US simulations. From the beginning, the antibodies were forced to dissociate from the binding mode with the WT/501Y.V2 RBD using SMD simulations. In particular, eight SMD trajectories were carried out to probe the most optimal-unbinding pathway. The trajectory, in which the rupture force, *F*_Max_, and pulling work, *W*, formed the smallest deviation in comparison with the median values, was used for generating US windows. In FPL, the antibody was pulled along *Z*-axis *via* an external force using cantilever *k* = 1000 kJ mol^−1^ nm^−2^ and constant velocity *v* = 0.001 nm ps^−1^. During the simulation, the RBD was softly fixed *via C*_α_ restraint. The pulling force, antibody displacement, and systemic coordinates were recorded every 33 integrated steps.

#### Umbrella sampling simulation

The systemic snapshots, which were extracted from the FPL trajectory since the antibody displaced every *ca.* 1.0 Å along the unbinding pathway *ξ*, were used as starting shapes of US simulations. *Ca.* 25 US windows each complex were simulated with a length of 10 ns of MD simulation to calculate the potential of mean force (PMF) curve. It should be noted that a short NPT simulation was executed to reduce initial fluctuations.^30,31^ The PMF values were calculated *via* the weighted histogram analysis method (WHAM).^32^ The free energy barriers, Δ*G*^++^_on_ and Δ*G*^++^_off_, and binding free energy, Δ*G*_b_, of the binding process between RBD and NAb were estimated as described as Fig. 1E.

### Analyzed tools

The free energy landscape (FEL) of the complex was constructed using the principal component analysis (PCA) method,^33^ in which coordinates PC1, first eigenvector, and PC2, second eigenvector, were calculated using GROMACS tools “gmx anaeig”. In particular, the PCs of backbone protein were calculated over the entire conformational ensemble. A non-bonded (NB) contact was counted when the pair between two heavy atoms is smaller than 4.5 Å. A hydrogen bond (HB) contact was counted when the angle ∠ between donor (D) – hydrogen (H) – acceptor (A) is larger than 135° and the distance between D and A is smaller than 3.5 Å. The PMF value was estimated *via* the Weighted Histogram Analysis Method (WHAM) with the execution of auto-correlated time. The computed error was calculated using the bootstrapping method.^34^ The solvent accessible surface area (SASA) was computed using GROMACS tool “gmx sasa”.

## Results and discussion

It should be noted that investigating structures of protein–protein complexes and understanding how they bind together are fundamental issues.^35^ Moreover, structures of several complexes remain difficult to solve experimentally.^36,37^ Furthermore, in order to characterize the protein–protein binding mechanisms, powerful experimental approaches are required,^38,39^ but the obtained data are normally limited or indirect. Obtaining direct data at an atomic level about binding pathways and physical insights into the binding mechanisms are still open issues.^35^ Atomistic MD simulations emerge as potential approaches for investigating both dynamics and structural change of protein–protein complexes.^17,40,41^ Using MD simulations, we can easily monitor the associate and dissociate processes of a monomer to the others.^28,42^ However, in fact, the association of two proteins may take place at a much longer time scale than unbiased simulations can usually reach. Normally, the enhanced sampling methods, which may combine several short simulation trajectories, are used to modeling the unbinding process of two proteins.^30,43^ The association of protein–protein is thus predicted.^43^ Therefore, in this work, atomistic simulations will be performed to reveal the insights at the atomic level of the binding process of an antibody to various SARS-CoV-2 variants RBD. Structural changes of the SARS-CoV-2 RBD + antibody complexes were characterized *via* MD simulations. Besides, because the glycan linked with the residue Asn343,^22^ which is far from the binding surface of RBD, the effect of the glycan on the binding of RBD and antibody can be thus neglected.^44^ Moreover, the obtained superposition of RBD with and without glycan confirmed the argument that the neglection of glycan probably adopts small effects on the interacted picture between RBD and antibody (Fig. S2 and S3 of the ESI file†). Thermodynamics and kinetics of the binding process were then revealed *via* a combination of SMD and US simulations.

Unbiased MD simulations were carried out to understand the structural change at the atomistic level of 501Y.V2 RBD + antibodies since the binding affinity of the antibodies to 501Y.V2 RBD was altered according to the recent report.^8,10,11,14^ The stabilized conformations of the RBD + antibody complexes were investigated over the equilibrium trajectories (*cf.* Fig. S4 of the ESI file†). Moreover, the obtained superposition of calculated metrics in the different intervals confirmed the convergence of unbiased MD simulations (Fig. S5–S8 of the ESI file†). Furthermore, the structural changes of the complexes were reported in Fig. S9 and S10 of the ESI file.† In particular, the backbone root-mean-square deviation (RMSD) and SASA of the complexes were enlarged when the 501Y.V2 variant were induced. However, the HB and NB contacts between RBD and NAb/fNAb were significantly reduced due to mutations. The obtained results imply that the protein–protein binding affinity between 501Y.V2 RBD to NAb/fNAb was decreased in comparison with WT one.

**Fig. 2:**
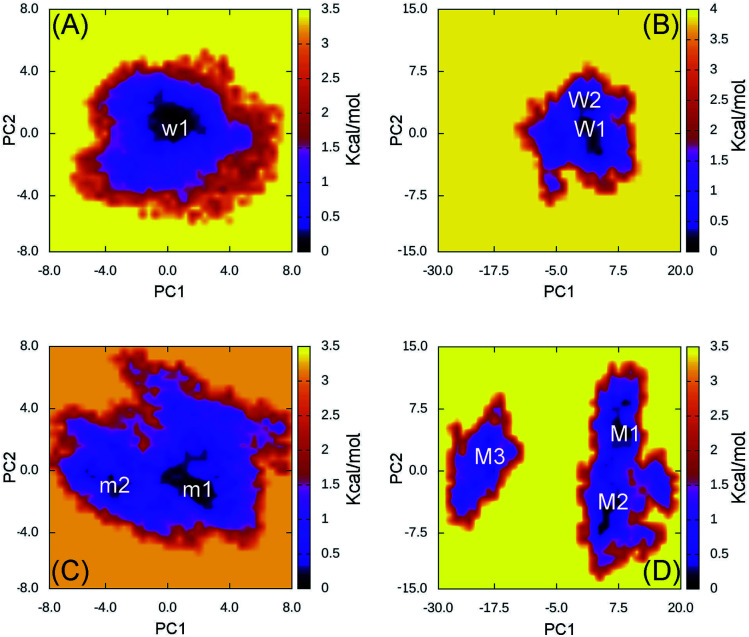
Free energy landscape of RBD + antibody complexes was constructed using PCA method. In particular, (A) presents the FEL of the WT RBD + fNAb over 4 independent MD trajectories; (B) mentions the FEL of the WT RBD + NAb over 4 independent MD trajectories; (C) describes the FEL of the 501Y.V2 RBD + fNAb over 4 independent MD trajectories; and (D) denotes the FEL of the 501Y.V2 RBD + NAb over 4 independent MD trajectories.

In order to estimate the representative conformations of the complexes, the two-dimensional FEL was generated using “gmx sham” tool.^33,45^ Two coordinates constructing FEL were first and second eigenvectors, which were computed using the PCA method.^33^ The obtained results were described in Fig. 2 (FEL for individual trajectories were presented in Fig. S11–S14 of the ESI†). Clearly, the 501Y.V2 variant increases the number of the FEL local minima implying that the 501Y.V2 complex is more flexible than the WT one. It also suggests that the binding free energy Δ*G*_b_ between 501Y.V2 RBD and NAbs is probably reduced. The obtained results are in good consistent with NB and HB contacts analyses above.

**Fig. 3:**
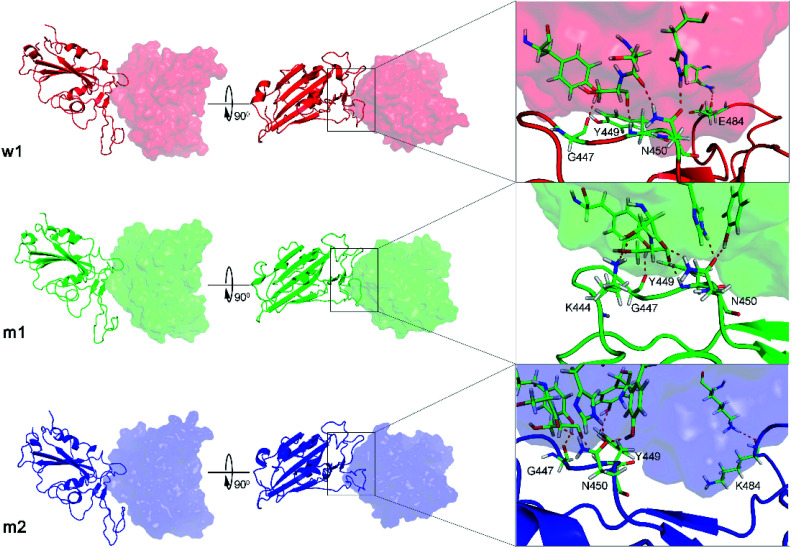
The representative structures of WT and 501Y.V2 RBD + fNAb in different perspective. The structures corresponds to the minima w1, m1, and m2, which were mentioned in Fig. 2. The structures were obtained using the clustering method with a backbone RMSD cut-off of 0.2 nm. The interaction diagram between RBD and fNAb was estimated using PyMOL tool.

The WT RBD + fNAb only formed one minimum noted as w1 in Fig. 2A, which is located at (CV1; CV2) coordinates of (0.40; 0.40). The representative structures of the complex corresponding to w1 was calculated using the clustering method with a backbone RMSD cutoff of 0.2 nm. In particular, the antibody adopted HBs to 4 residues of the WT RBD including G447, Y449, N450, and E484 (*cf.*Fig. 3). These results suggest that a mutation E484K will significantly alter the binding affinity/mechanism of the RBD + fNAb. Two minima were observed in FEL of 501Y.V2 RBD + fNAb, which are located at (CV1; CV2) coordinates of (1.60; −1.40) and (−3.60; −1.00) denoted as m1 and m2, respectively. The corresponding population of m1 and m2 is 75 and 25%, respectively. Analyzing the representative structure m1, the antibody was found to be able to form HBs to the residues K444, G447, Y449, and N450 of the 501Y.V2 RBD. The corresponding residues of m2, which formed HBs to RBD 2–4, are G447, Y449, N450, and K484 (*cf.*Fig. 3). The observed structural changes imply that the binding affinity and kinetics between RBD and fNAb probably change. A similar story of RBD + NAb, which is mentioned in detail below, was obtained and confirmed the results.

**Fig. 4:**
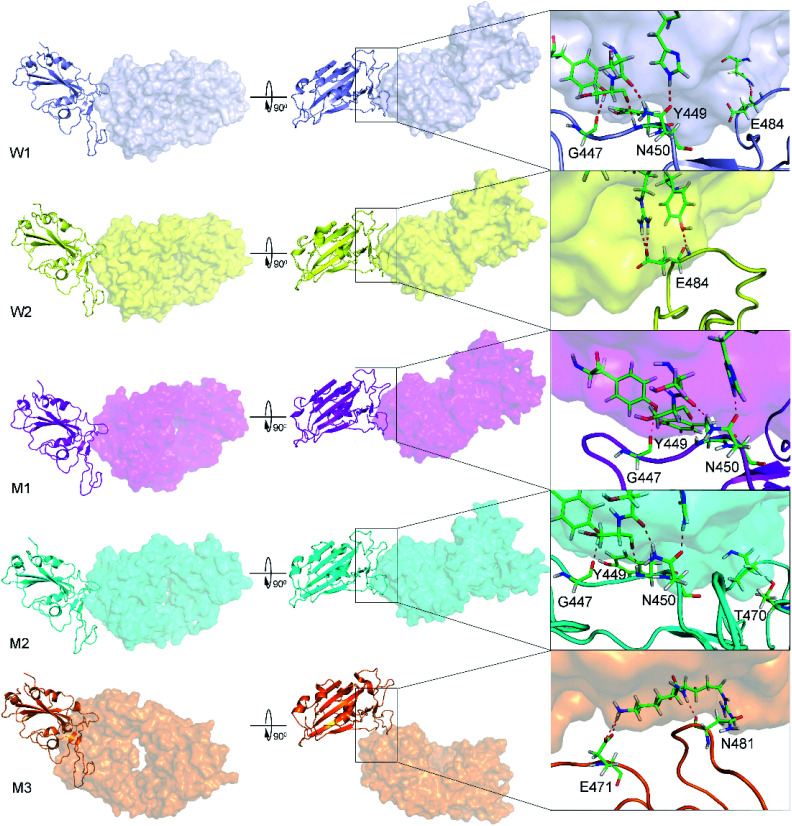
The representative structures of WT and 501Y.V2 RBD + NAb corresponding to the minima W1, W2, M1, M2, and M3, which were mentioned in Fig. 2. The structures were obtained using the clustering method with a backbone RMSD cut-off of 0.2 nm. The interaction diagram between RBD and NAb were obtained using PyMOL tool.

The WT/501Y.V2 RBD + NAb systems were also investigated. FEL of the complexes was significantly altered when the mutations were induced. The WT RBD + NAb formed two minima, which were shown in Fig. 2B. These minima located at (CV1; CV2) coordinates of (0.63; 0.75) and (0.63; 3.38) denoting as W1 and W2, respectively. The corresponding population of W1 and W2 is 63 and 37%, respectively. Besides that, the 501Y.V2 RBD + NAb FEL (Fig. 2D) adopted three minima, which located at (CV1; CV2) coordinates of (7.50; 4.13), (5.63; −4.50), and (−18.8; 1.50) labelling as M1, M2, and M3, respectively. The corresponding population of M1, M2, and M3 is 45, 38, and 17%, respectively. Analyzing the complex W1, the HBs were observed between antibody and residues G447, Y449, N450, and E484 of the RBD that is in good consistency to the w1 case. However, HBs were only found between the NAb and residue E484 of the WT RBD in the complex W2 (Fig. 4). The obtained results indicate that residue E484 plays an important role in the binding process of the antibody to the RBD that is in good consistent with the recent work.^13^ Replacing the E484 with another residue probably modifies the binding mechanism of the antibodies to RBD rather than substitutions at the different positions. Moreover, it should be noted that in the 501Y.V2 variant induced, a lysine residue substitutes the glutamate residue at the sequence 484. The replacement probably terminates the HBs and weakening the attracted force between the NAb and the RBD. The argument was confirmed *via* evaluations of the representative structures of 501Y.V2 RBD + NAb complexes. In conformation M1, the HBs between NAb and the residues G447, Y449, and N450 of RBD were found. The residues G447, Y449, N450, and T470 of 501Y.V2 RBD procedure HBs to NAb in conformation M2. Furthermore, the NAb only found two HBs to the residue E471 and N481 of the 501Y.V2 RBD. The free energy approach should be carried out to clarify the change of binding affinity upon the structural changes of the 501Y.V2 RBD + NAb complexes.

As discussion above, the RBD + fNAb structure is more flexible when the 501Y.V2 variant was induced. The binding affinity/mechanism of the complex are thus altered. In this work, a combination of SMD/US simulations were carried out to probe the change in RBD + NAbs association. The SMD was used to generate US windows (*cf.* the ESI file†). The free energy profile was then calculated using the WHAM.^32^ The binding free energy Δ*G*_b_ between RBD and NAbs is able to calculate *via* PMF curve as mentioned in Fig. 1E.^30,31^ Moreover, the free energy barriers Δ*G*^++^_on_ and Δ*G*^++^_off_, which were associated with the binding kinetic rate constant *k*_on_ and the unbinding kinetic rate constant *k*_off_ can be also estimated, respectively.

The calculated results for free energy barriers (*cf.*Table 1) indicated that the NAbs will bind to 501Y.V2 RBD more difficult than WT one because of the larger Δ*G*^++^_on_. NAbs are much easier to bind to than to unbind from RBD, because the Δ*G*^++^_off_ is larger than the Δ*G*^++^_on_. However, in the M3 case, the Δ*G*^++^_off_ = 0.14 ± 0.18 kcal mol^−1^ is significantly smaller than the Δ*G*^++^_on_ = 2.83 ± 0.65 kcal mol^−1^ indicating that it takes more time for NAb to bind to 501Y.V2 RBD for them unbind. Moreover, the observations were also supported by the binding free energy, Δ*G*_b_, calculations, in which the thermodynamic metric corresponding to the association between NAbs and RBD is significantly decreased when the 501Y.V2 variant was induced (Table 1). The NAb is thus resisted to bind to 501Y.V2 RBD. Therefore, it may be argued that the 501Y.V2 variant could reduce the vaccine efficiency. The observation is in good agreement with the experimental data that the neutralizing antibody is weaker bind to 501Y.V2 spike protein than to WT one.^8,10,11,14^ However, besides, the free energy value over the population of minima and the predicted kinetic coefficients were reported in Table S2 of the ESI file.† The predicted values should only use for qualitative comparisons between the RBD + antibody complexes, because the absolute value of the metrics is quite different from experimental values.^46^

**Table 1:** The calculated results using SMD and US simulations^a^

No.	System	*F*_Max_	*W*	Δ*G*^++^_on_	Δ*G*^++^_off_	Δ*G*_b_
1	WT RBD + fNAb (w1)	1388.0 ± 18.6	139.9 ± 3.3	0.24 ± 0.20	18.31 ± 0.82	−18.07 ± 0.84
2	501Y.V2 RBD + fNAb (m1)	859.3 ± 40.7	72.8 ± 3.4	0.81 ± 0.26	12.00 ± 0.82	−11.19 ± 0.77
3	501Y.V2 RBD + fNAb (m2)	1007.4 ± 31.2	86.8 ± 3.1	0.73 ± 0.11	11.62 ± 0.54	−10.89 ± 0.55
4	WT RBD + NAb (W1)	1133.6 ± 39.0	178.8 ± 8.5	0.36 ± 0.75	39.82 ± 1.31	−39.46 ± 1.08
5	WT RBD + NAb (W2)	1137.6 ± 25.8	181.5 ± 6.5	0.76 ± 0.29	43.36 ± 0.73	−42.60 ± 0.67
6	501Y.V2 RBD + NAb (M1)	745.5 ± 25.4	96.1 ± 3.6	0.62 ± 0.15	21.64 ± 0.66	−20.93 ± 0.68
7	501Y.V2 RBD + NAb (M2)	748.0 ± 33.1	81.6 ± 6.5	0.42 ± 0.24	16.16 ± 0.88	−15.74 ± 0.91
8	501Y.V2 RBD + NAb (M3)	470.2 ± 25.7	50.5 ± 4.5	0.14 ± 0.18	2.83 ± 0.65	−2.70 ± 0.68

a The calculated results over SMD and US simulations. The details of free energy profile and histograms over US simulations were reported in Fig. S15–S18 of the ESI file.

**Fig. 5:**
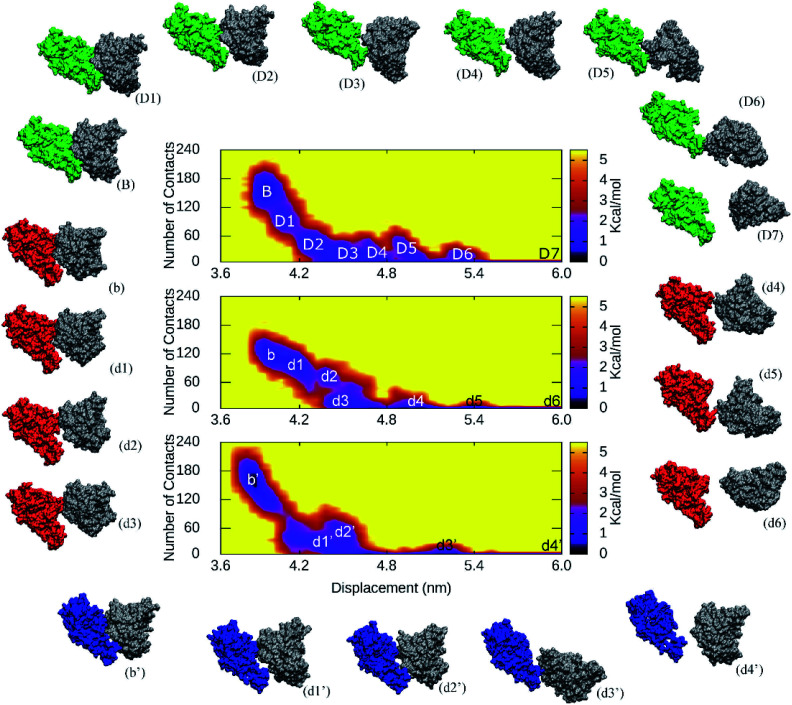
The collective-variable FEL revealed the unbinding pathways of fNAb from the binding mode with WT/501Y.V2 RBD over US simulations. The representative structures of complexes were also estimated using the clustering method with a backbone RMSD cutoff of 0.2 nm.

The collective-variable FEL,^47^ was constructed by number of contacts between two proteins within 0.45 nm and the displacement of the antibody, revealed the unbinding pathway of NAbs. The obtained FEL was shown in Fig. 5 and S19 of the ESI file.† The representative structures of the complexes within a backbone RMSD of 0.2 nm were then estimated using clustering method.^33^ The unbinding pathways were significantly altered under effects of the 501Y.V2 variant. A larger number of transition states of the WT RBD + fNAb complex implies that it is hard to unbind the antibody from WT system than 501Y.V2 variant. Moreover, the representative structures B, b, and b′ correspond to the binding model of the RBD + fNAb complexes. The structures D7, d6, and d4′ respond to the minima where the fNAb completely detached from RBD. The other conformations correspond to dissociated structures along unbinding pathways. The similar picture was also observed when the RBD + NAb complexes were investigated (Fig. S19 of the ESI file†).

## Conclusions

In this work, the NAb resistance of 501Y.V2 variant was investigated using atomistic simulations. In particular, the binding pose of NAb/fNAb to WT/501Y.V2 RBD was revealed using atomistic simulations. Reducing number of HB and NB contacts between RBD and antibodies were observed when the 501Y.V2 variant was induced. Increasing FEL minima of 501Y.V2 RBD + NAb/fNAb in comparison with the WT RBD systems infer that the complex 501Y.V2 RBD + NAb/fNAb is more unstable than the WT one. Moreover, thermodynamics and kinetics of the binding process between RBD and NAb were also determined using SMD/US simulations. Interestingly, the binding free energy Δ*G*_b_ of WT RBD + NAb/fNAb is significantly smaller than that of 501Y.V2 RBD + NAb/fNAb. It is consistent with the results of the binding kinetic rate constant *k*_on_ and the unbinding kinetic rate constant *k*_off_. Poorly binding affinity of NAb/fNAb to 501Y.V2 RBD confirms the antibody resistance of the South African variant.^8,10,11,14^ Furthermore, the RBD + fNAb system can be used as an affordable model to investigate the change of the binding process between mutations RBD and antibodies. The required computing resources are thus reduced significantly.

## Conflicts of interest

There are no conflicts to declare.

## Supplementary Material

RA-011-D1RA04134G-s001
