# Supplementary material for: Thermodynamics and kinetics in antibody resistance of the 501Y.V2 SARS-CoV-2 variant
Source: RSC Adv. 2021 Oct 13;11(53):33438–46. doi: 10.1039/d1ra04134g (PMC9042284; doi:10.1039/d1ra04134g)
Supplement: RA-011-D1RA04134G-s001 [file RA-011-D1RA04134G-s001.pdf]

Electronic Supplementary Material (ESI) for RSC Advances.
This journal is © The Royal Society of Chemistry 2021

Supporting information

Thermodynamics and Kinetics in Antibody Resistance of the 501Y.V2 SARS-CoV-2 Variant

Son Tung Ngo,[ab*] Trung Hai Nguyen,[ab] Duc-Hung Pham,[c] Nguyen Thanh Tung,[de] and Pham Cam Nam,[f*]

[a]Laboratory of Theoretical and Computational Biophysics, Ton Duc Thang University, Ho Chi Minh City 700000, Vietnam

[b]Faculty of Applied Sciences, Ton Duc Thang University, Ho Chi Minh City 700000, Vietnam

[c]Division of Immunobiology, Hepatology and Nutrition Cincinnati Children's Hospital Medical Center, Cincinnati 45229, OH, USA

[d]Institute of Materials Science, Vietnam Academy of Science and Technology, Hanoi 100000, Vietnam

[e]Graduate University of Science and Technology, Vietnam Academy of Science and Technology, Hanoi 100000, Vietnam

[f]Department of Chemical Engineering, The University of Da Nang, University of Science and Technology, Da Nang City 550000, Vietnam

*Email: ngosontung@tdtu.edu.vn and pcnam@dut.udn.vn

Table S1. Systemic Configuration Information during unbiased and biased MD Simulations.

System	Simulation Method	Box Type	Box Size	Volume (nm^3)	Total Atoms
WT RBD + FAP 2-4	MD	Dodecahedron	(11.51, 11.51, 11.51)	971.33	106 209
501Y.V2 RBD + FAP 2-4	MD	Dodecahedron	(11.51, 11.51, 11.51)	971.33	106 231
WT RBD + FAP	MD	Dodecahedron	(15.10, 15.10, 15.10)	2391.18	232 378
501Y.V2 RBD + FAP	MD	Dodecahedron	(15.10, 15.10, 15.10)	2391.18	232 376
WT RBD + FAP 2-4 (**w1**)	SMD/US	Rectangular	(7.64, 8.08, 14.80)	913.62	88 128
501Y.V2 RBD + FAP 2-4 (**m1**)	SMD/US	Rectangular	(7.64, 8.08, 14.80)	913.62	88 138
501Y.V2 RBD + FAP 2-4 (**m2**)	SMD/US	Rectangular	(7.64, 8.08, 14.80)	913.62	88 149
WT RBD + FAP 2-4 (**W1**)	SMD/US	Rectangular	(8.57, 9.04, 17.31)	1341.05	132 409
WT RBD + FAP 2-4 (**W2**)	SMD/US	Rectangular	(8.57, 9.04, 17.31)	1341.05	132 592
501Y.V2 RBD + FAP (**M1**)	SMD/US	Rectangular	(8.57, 9.04, 17.31)	1341.05	132 395
501Y.V2 RBD + FAP (**M2**)	SMD/US	Rectangular	(8.57, 9.04, 17.31)	1341.05	132 464
501Y.V2 RBD + FAP (**M3**)	SMD/US	Rectangular	(8.57, 9.04, 17.31)	1341.05	132 320

Table 2. The calculated results using SMD and US simulations.[a]

N°	System	$\Delta G_{\mathrm{on}}^{++}$ (kcal mol^{-1})	k_{on} (M^{-1} s^{-1})	$\Delta G_{\mathrm{off}}^{++}$ (kcal mol^{-1})	k_{off} (s^{-1})	ΔG_{b} (kcal mol^{-1})	Predicted k_{D} range
1	WT RBD + fNAb	0.24	7.25×10^{-12}	18.31	7.38×10^{-1}	-18.07	Sub-Picomolar
2	501Y.V2 RBD + fNAb	0.79	2.96×10^{-12}	11.91	2.48×10^{4}	-11.12	High-Nanomolar
3	WT RBD + NAb	0.51	4.69×10^{-12}	41.13	5.47×10^{-17}	-40.62	High-Quectomolar
4	501Y.V2 RBD + NAb	0.46	5.05×10^{-12}	16.36	1.76×10^{1}	-15.86	Picomolar

[a]The calculated results were obtained over the population of the minima.

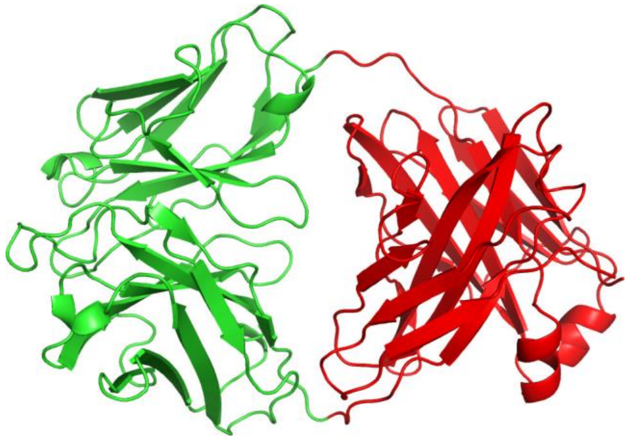

Figure S1. The structure of neutralizing antibody. In particular, the fNAb, which residue ranges from 1-127 (chain A) and 1-110 (chain B), is noted with green color.

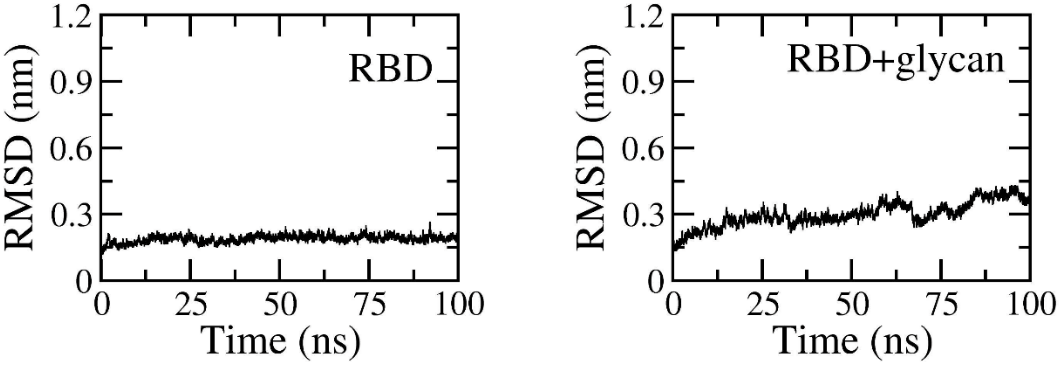

Figure S2. Backbone RMSD of RBD and RBD + glycan during MD simulations.

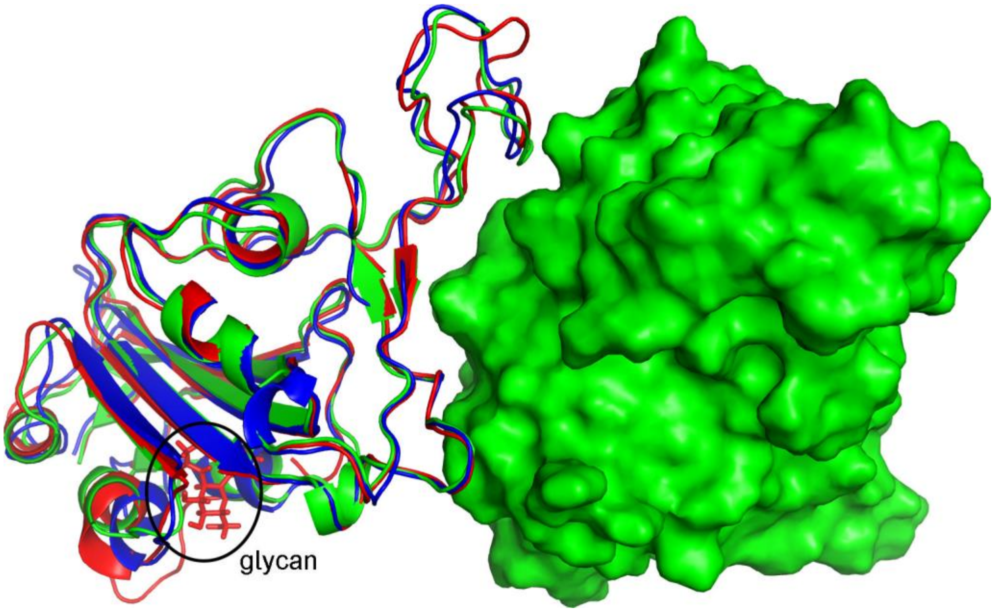

Figure S3. Superposition of the representative structure of RBD + glycan (red) and RBD (blue), which were clustered from interval 40-100 ns. The complex RBD + fNAb (initial conformation) was also prepared to confirm the superposition of the RBD with and without the glycan.

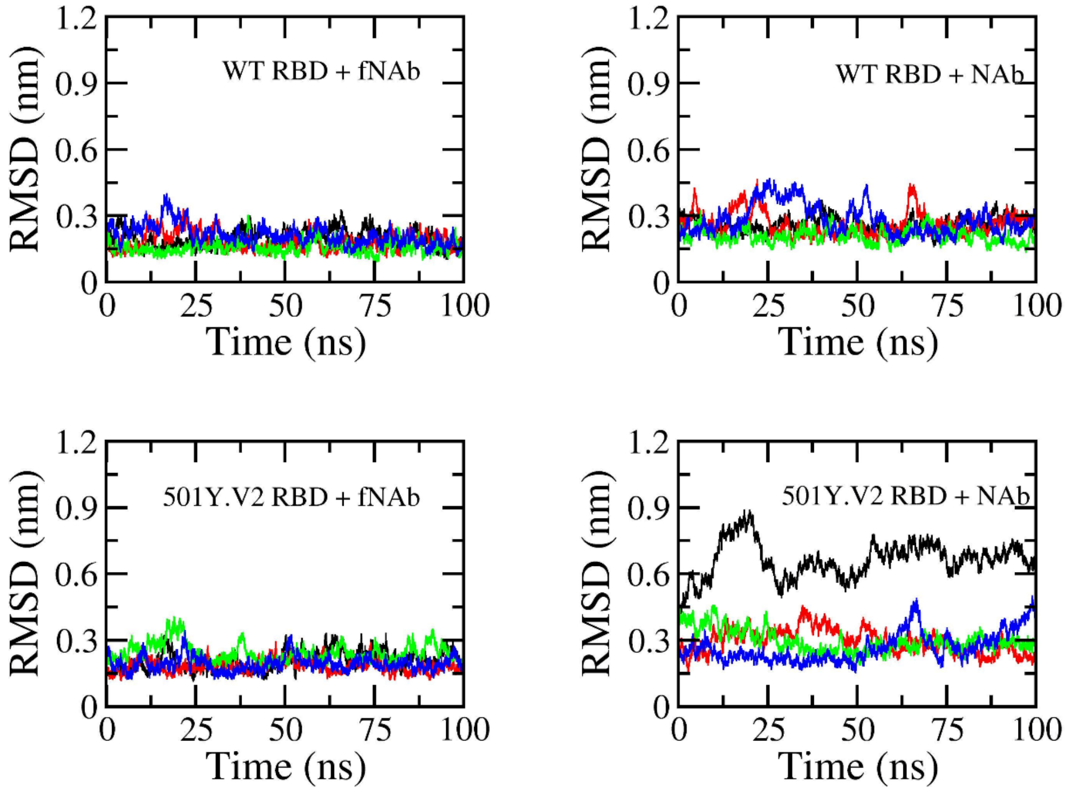

Figure S4. Backbone RMSD of RBD + antibody systems during 4 independent MD simulations.

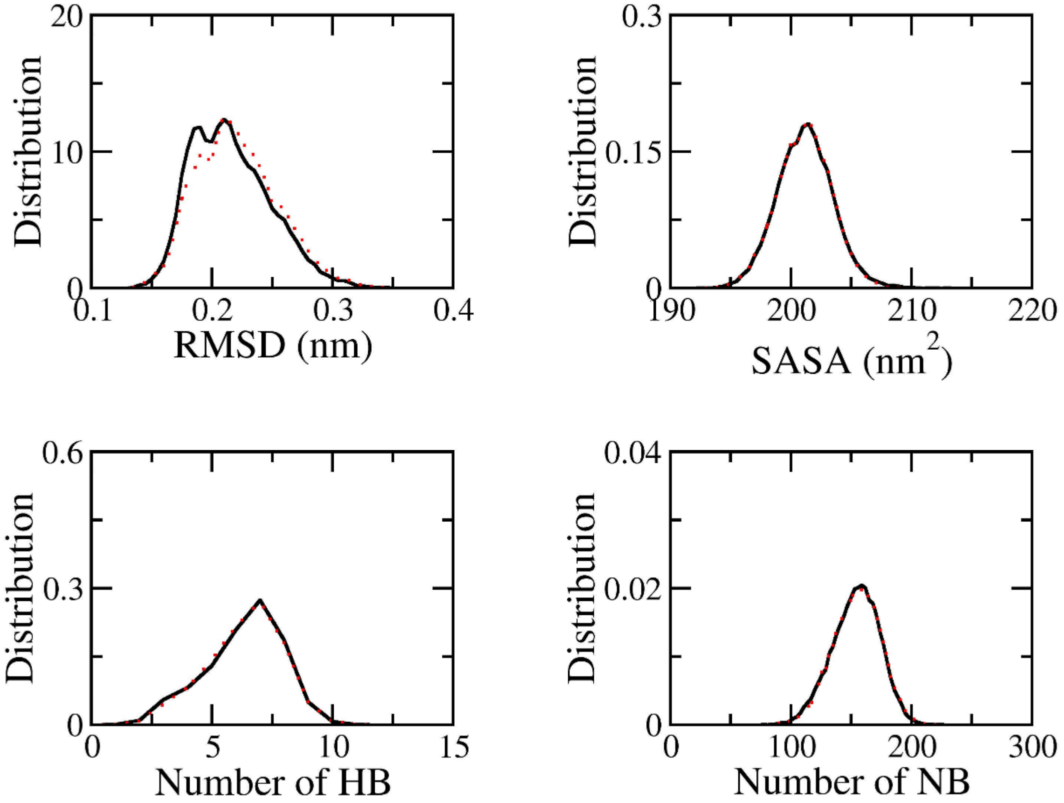

Figure S5. The superposition between computed metrics of WT RBD + fNAb in different intervals including 40-100 ns (black curve) and 40-80 ns (dotted red line) of MD simulations. The results were obtained since combined four independent MD trajectories.

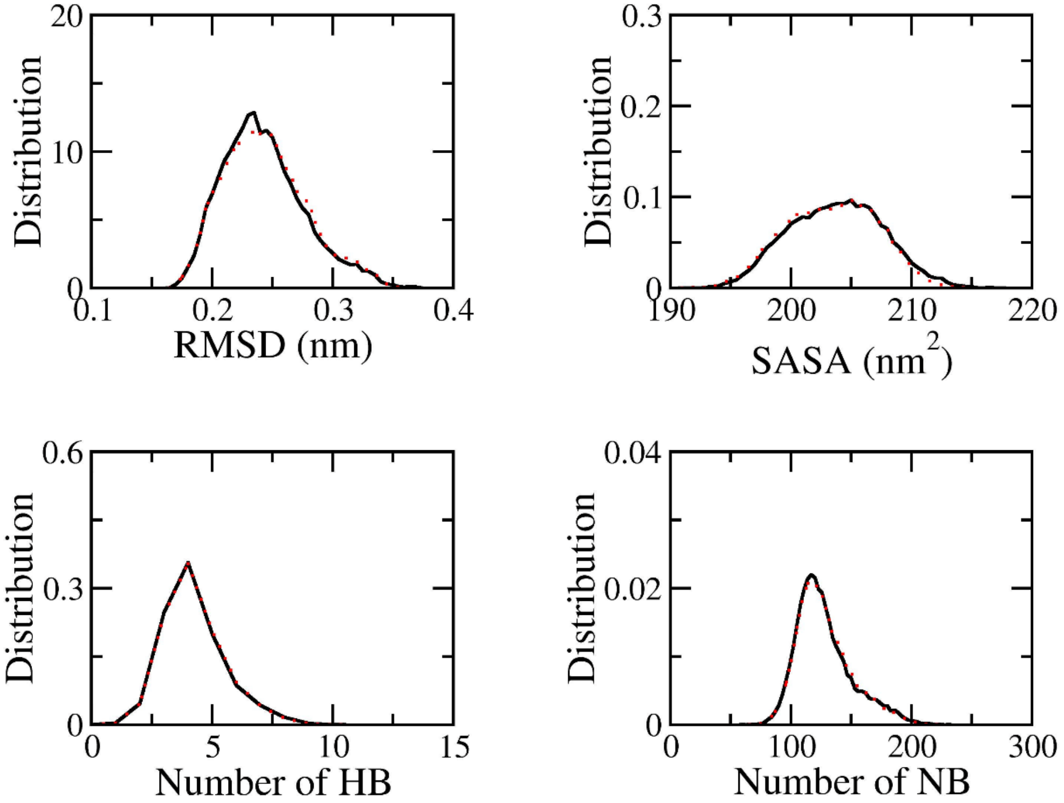

Figure S6. The superposition between computed metrics of 501Y.V2 RBD + fNAb in different intervals including 40-100 ns (black curves) and 40-80 ns (dotted red line) of MD simulations. The results were obtained since combined four independent MD trajectories.

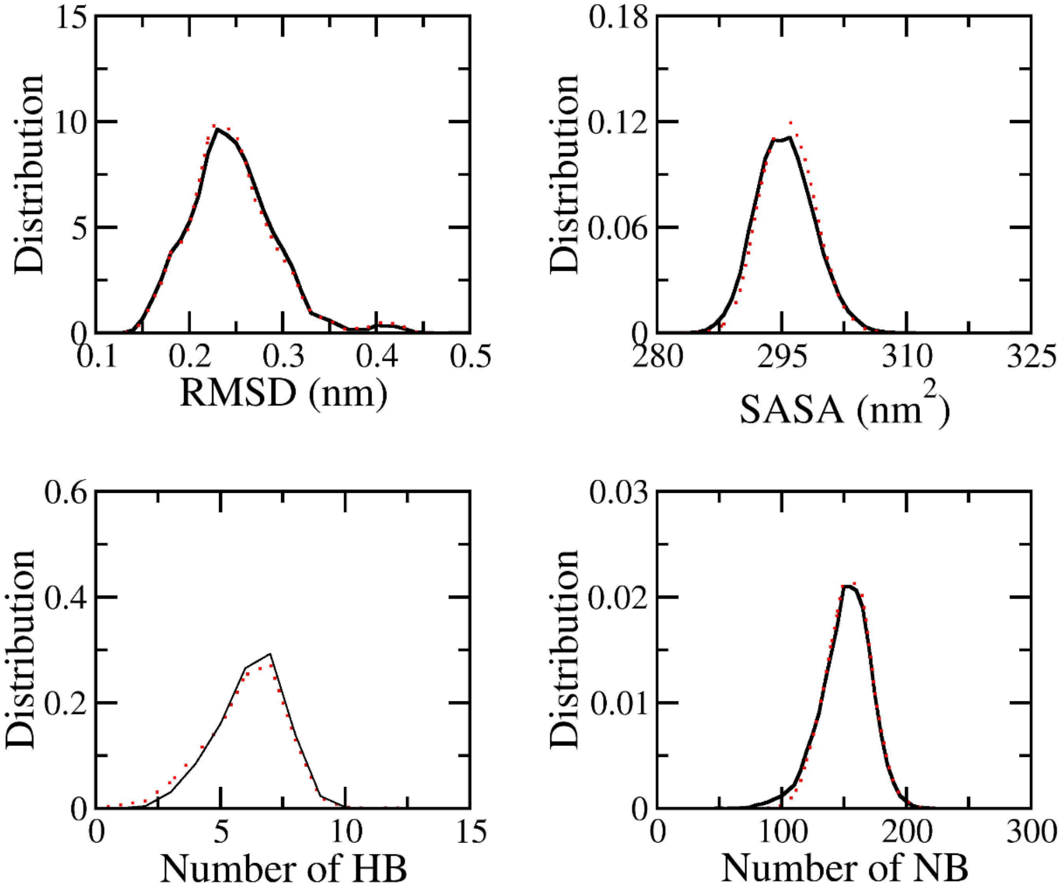

Figure S7. The superposition between computed metrics of WT RBD + NAb in different intervals including 40-100 ns (black curve) and 40-80 ns (dotted red line) of MD simulations. The results were obtained since combined four independent MD trajectories.

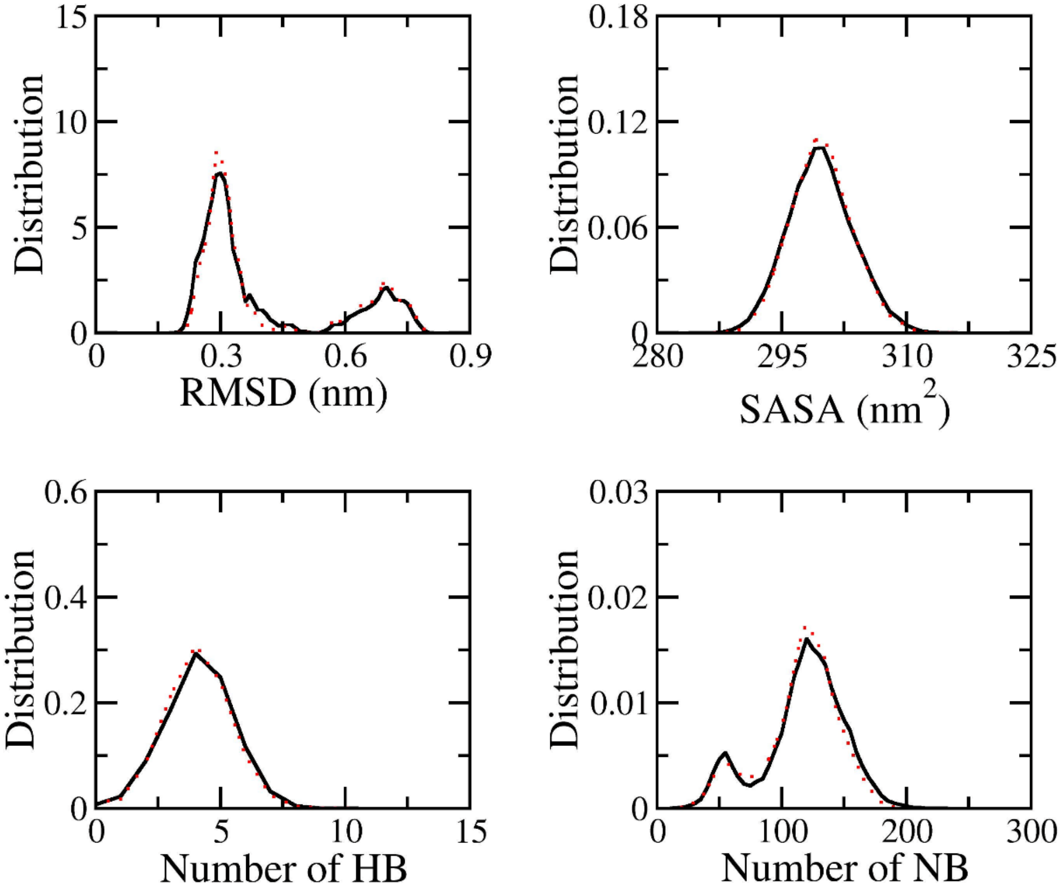

Figure S8. The superposition between computed metrics of 501Y.V2 RBD + NAb in different intervals including 40-100 ns (black curve) and 40-80 ns (dotted red line) of MD simulations. The results were obtained since combined four independent MD trajectories.

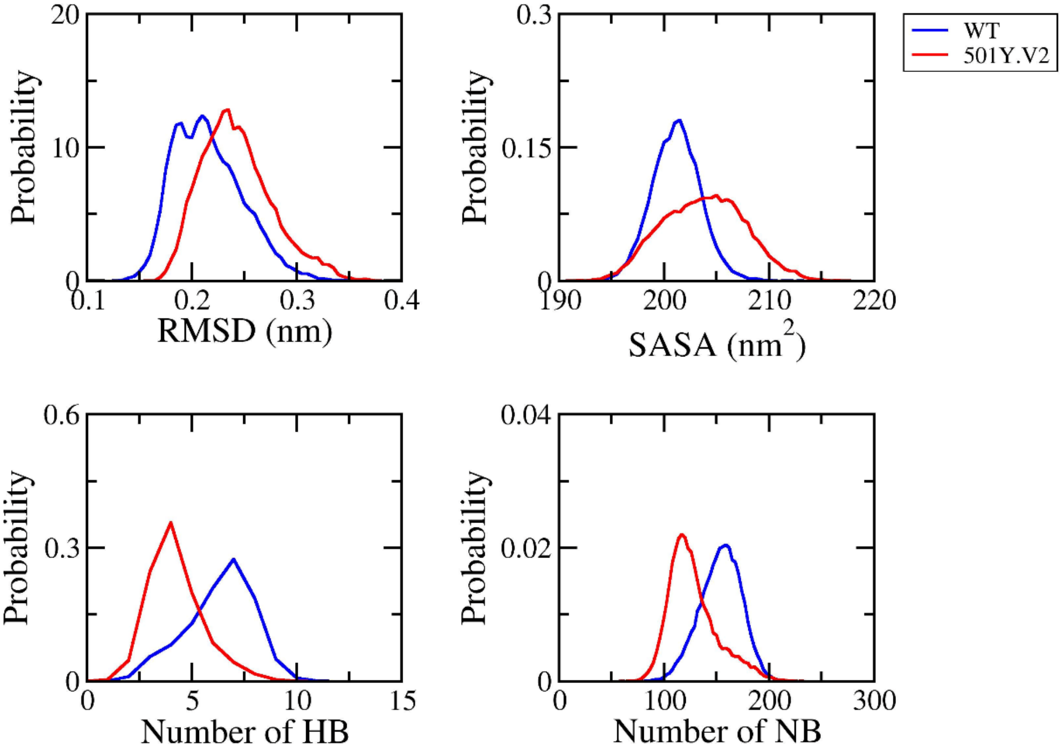

Figure S9. The difference between computed metrics of WT and 501Y.V2 RBD + fNAb.

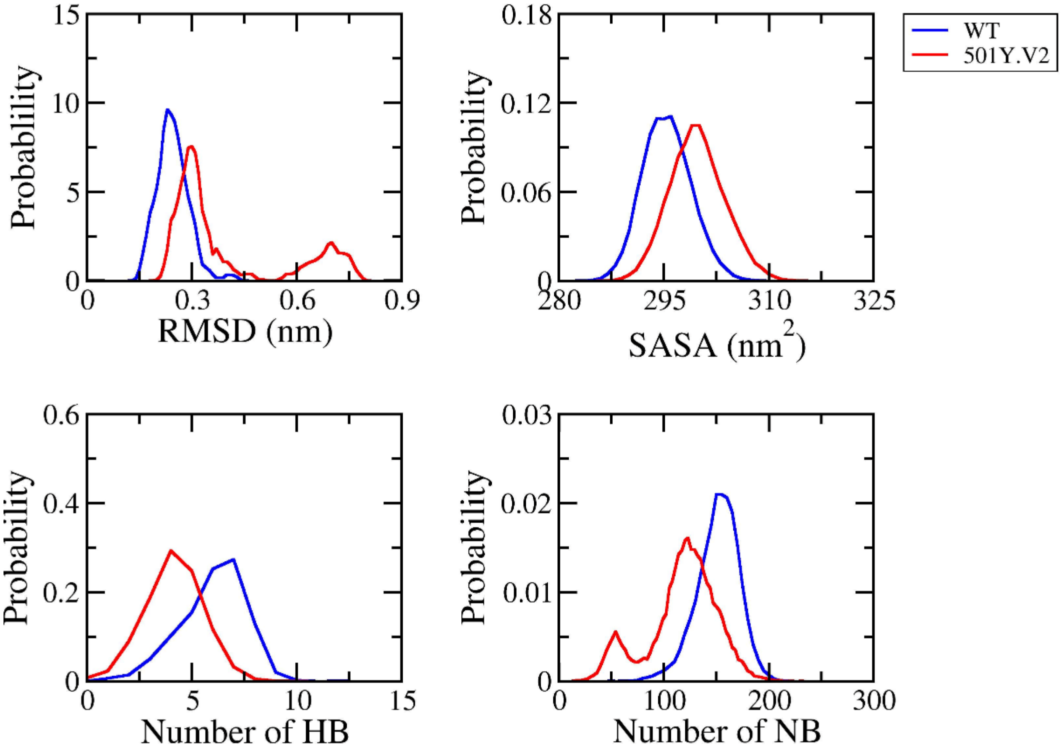

Figure S10. The difference between computed metrics of WT and 501Y.V2 RBD + NAb.

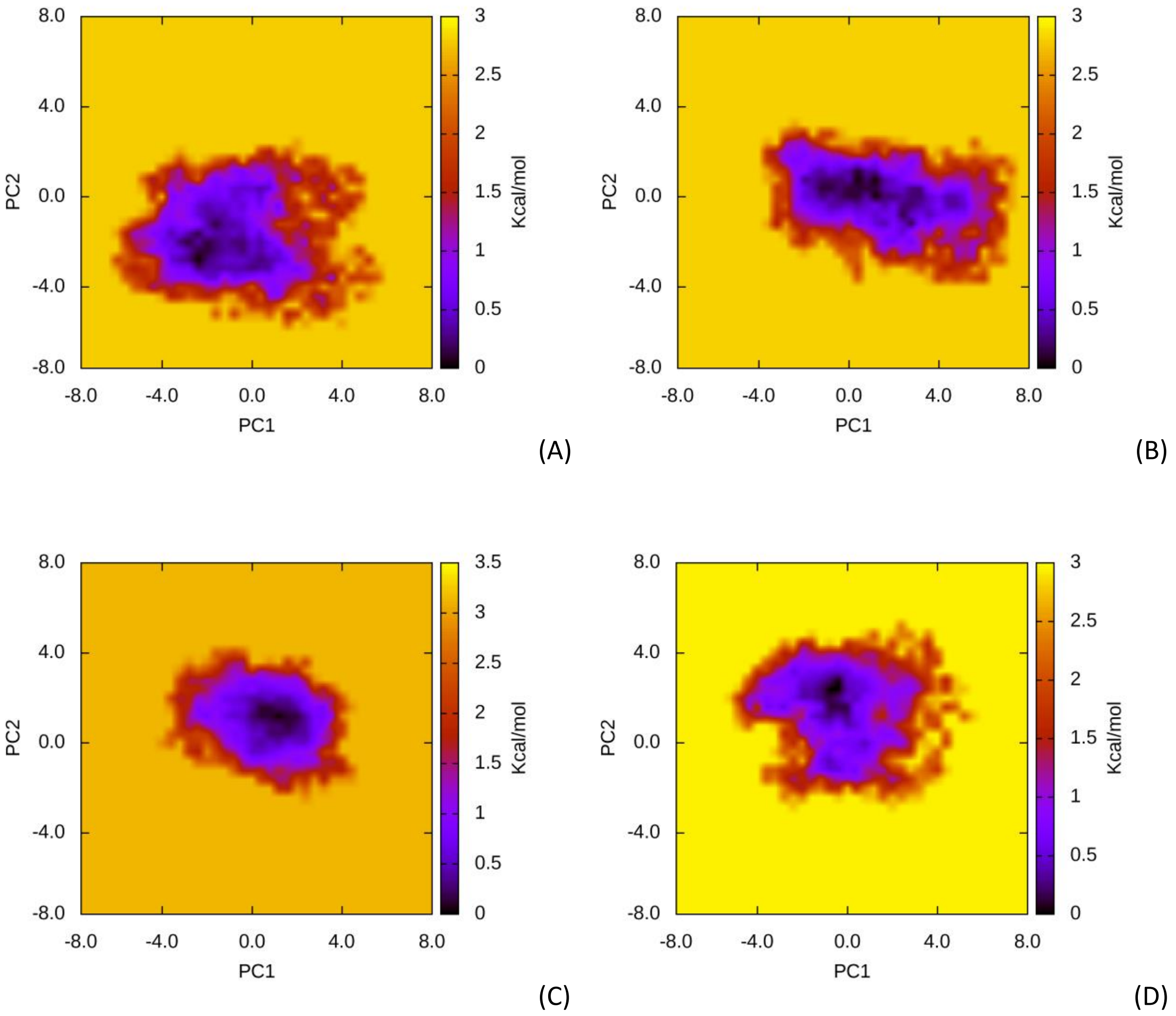

Figure S11. Free energy landscape of WT RBD + fNAb complexes was constructed using PCA method. In particular, (A) presents the FEL of trajectory 1, (B) presents the FEL of trajectory 2, (C) presents the FEL of trajectory 3, (D) presents the FEL of trajectory 4.

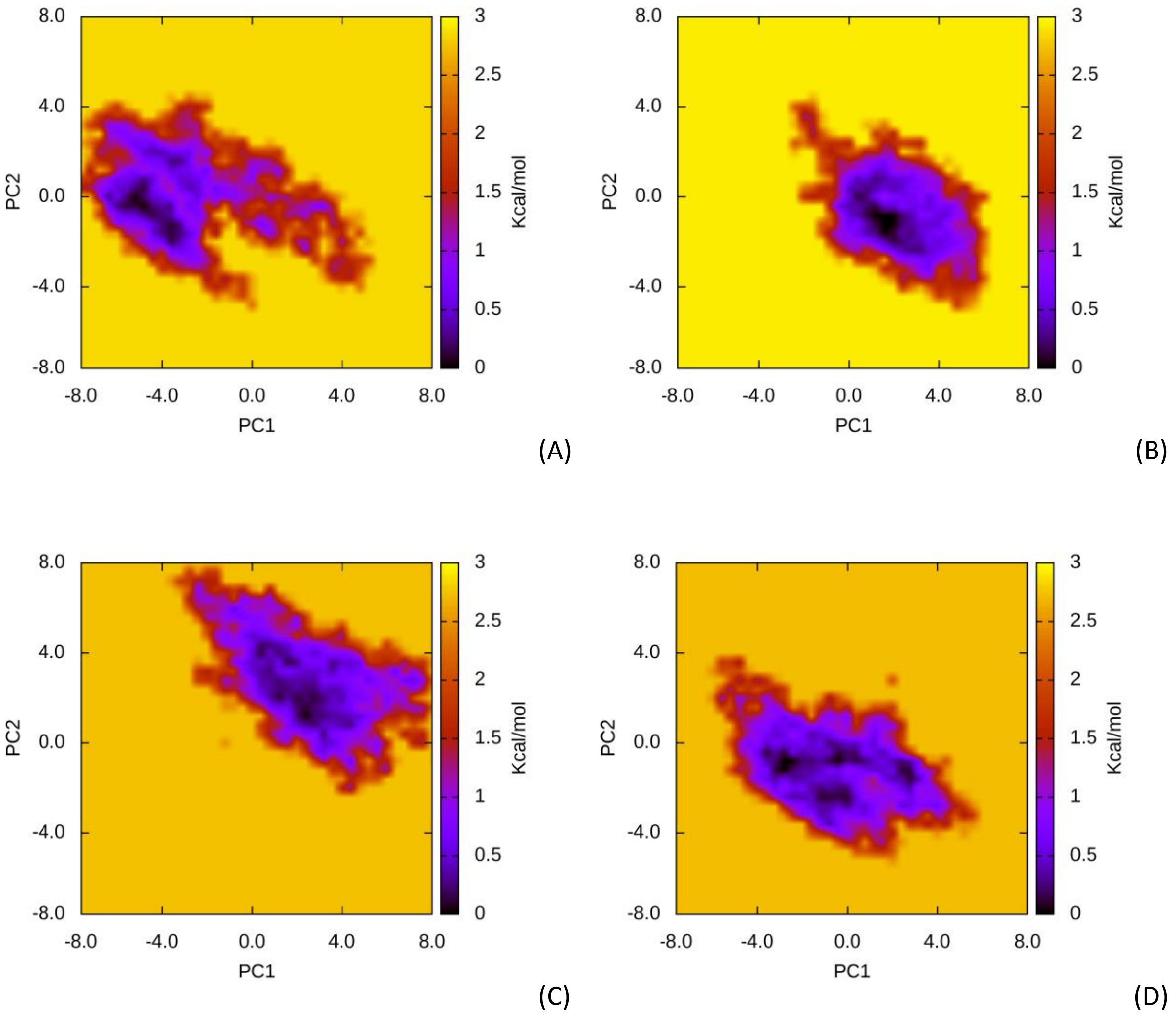

Figure S12. Free energy landscape of 501Y.V2 RBD + fNAb complexes was constructed using PCA method. In particular, (A) presents the FEL of trajectory 1, (B) presents the FEL of trajectory 2, (C) presents the FEL of trajectory 3, (D) presents the FEL of trajectory 4.

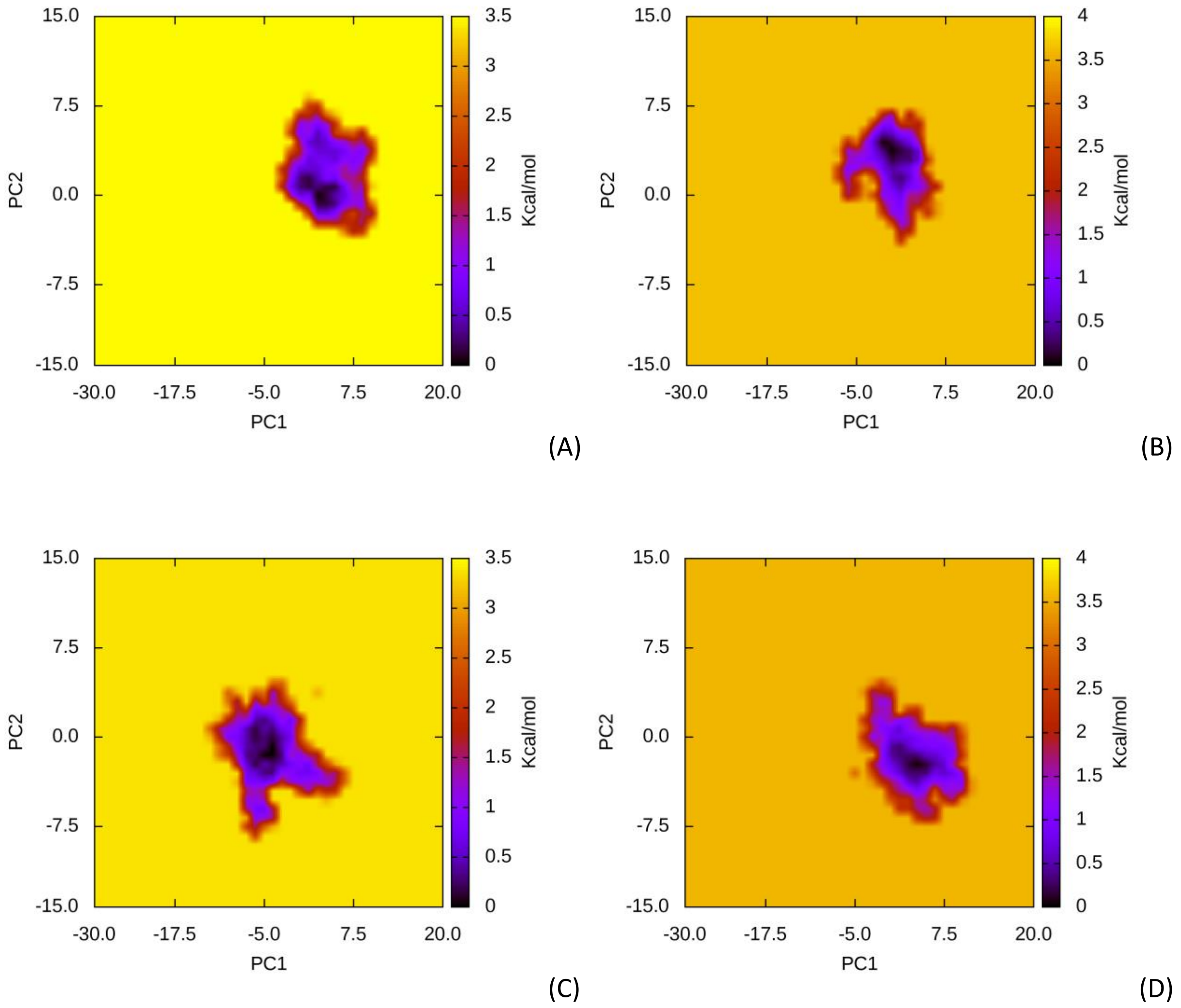

Figure S13. Free energy landscape of WT RBD + NAb complexes was constructed using PCA method. In particular, (A) presents the FEL of trajectory 1, (B) presents the FEL of trajectory 2, (C) presents the FEL of trajectory 3, (D) presents the FEL of trajectory 4.

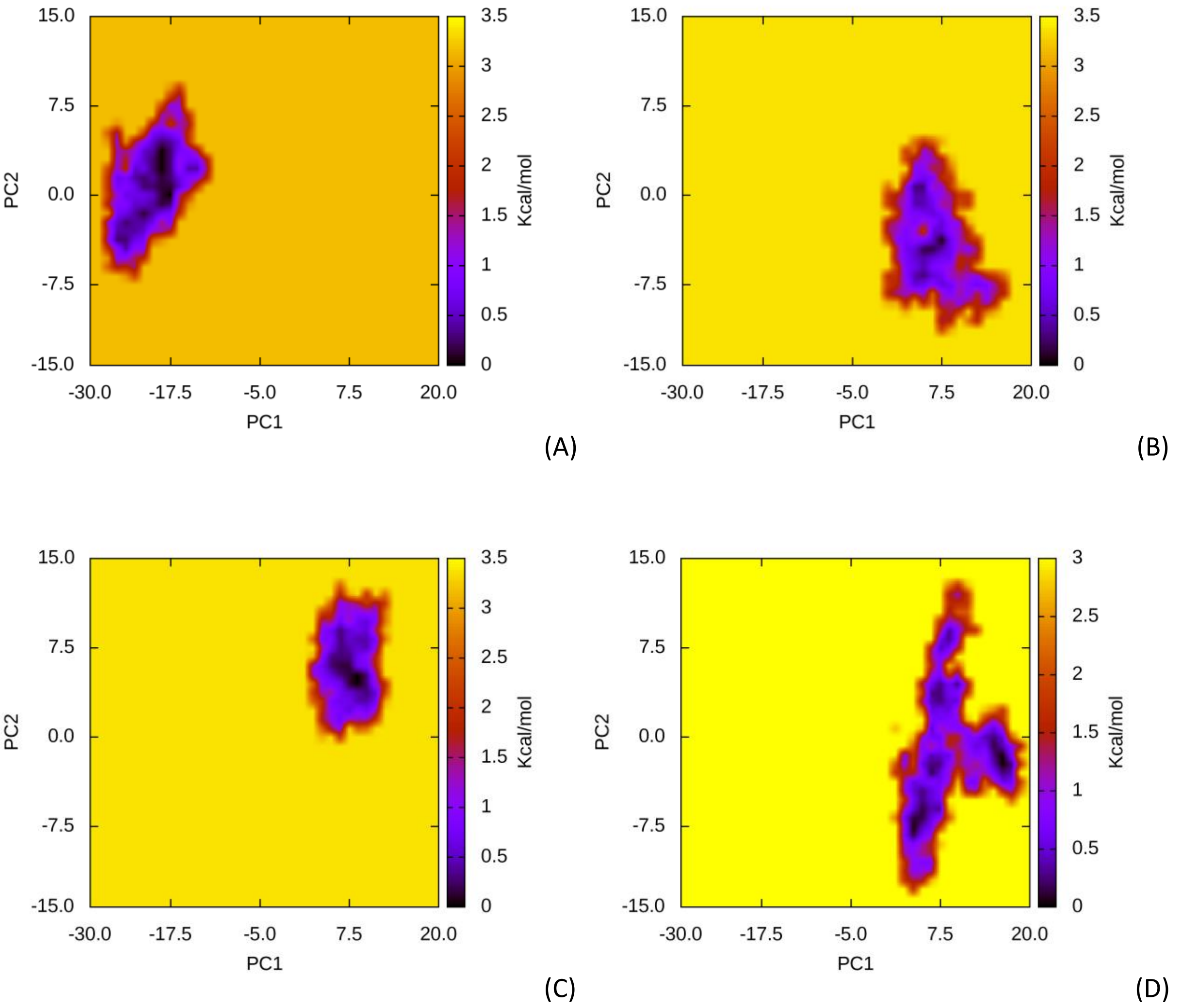

Figure S14. Free energy landscape of 501Y.V2 RBD + NAb complexes was constructed using PCA method. In particular, (A) presents the FEL of trajectory 1, (B) presents the FEL of trajectory 2, (C) presents the FEL of trajectory 3, (D) presents the FEL of trajectory 4.

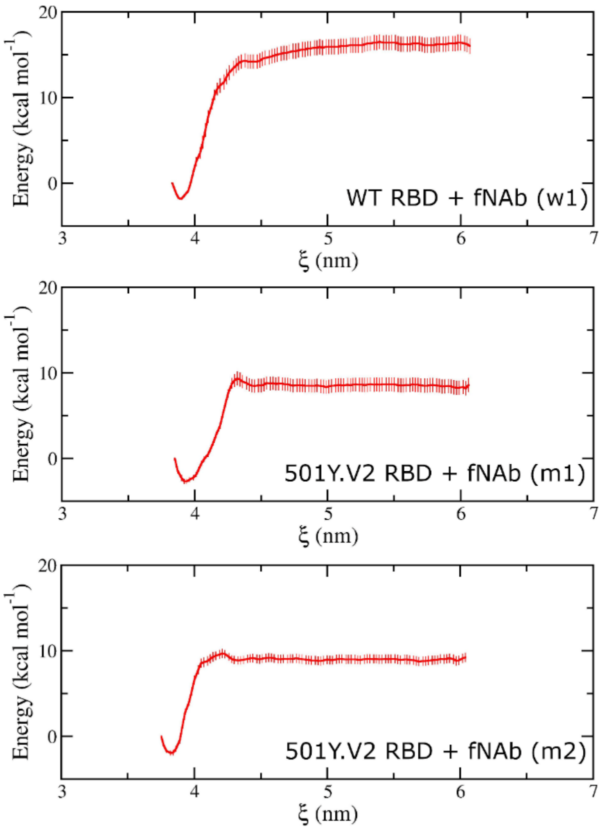

Figure S15. Free energy profile over the unbinding process of fNAb out of the WT/501Y.V2 RBD + fNAb complexes. The results were obtained using the WHAM calculation.

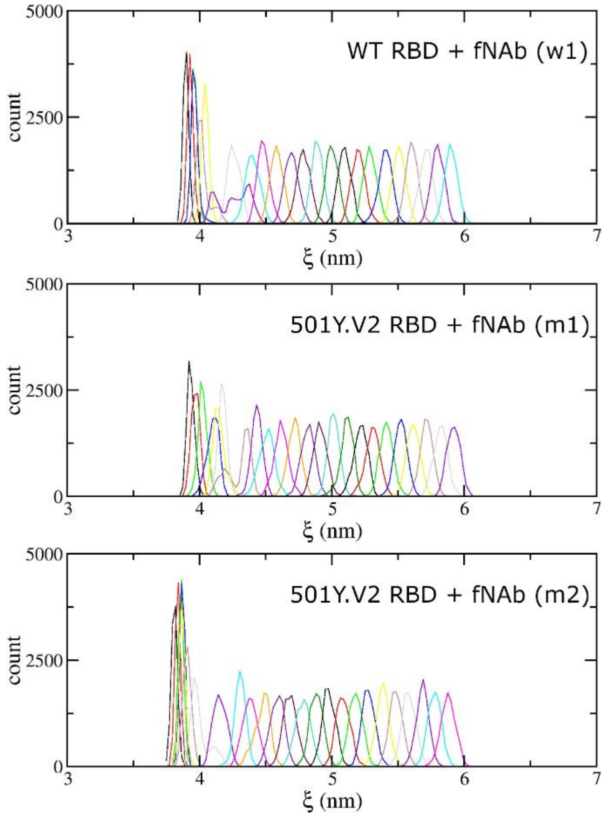

Figure S16. The histograms of US simulations over the unbinding process of fNAb out of the WT/501Y.V2 RBD + fNAb complexes.

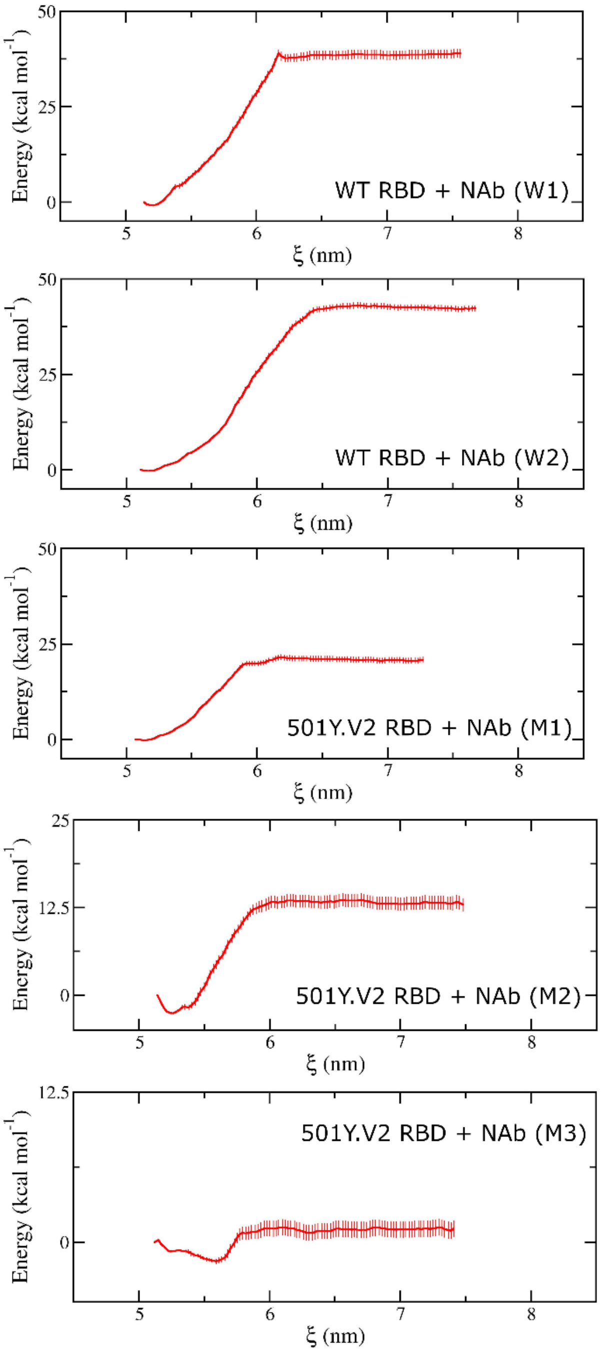

Figure S17. Free energy profile over the unbinding process of NAb out of the WT/501Y.V2 RBD + NAb complexes. The results were obtained using the WHAM calculation.

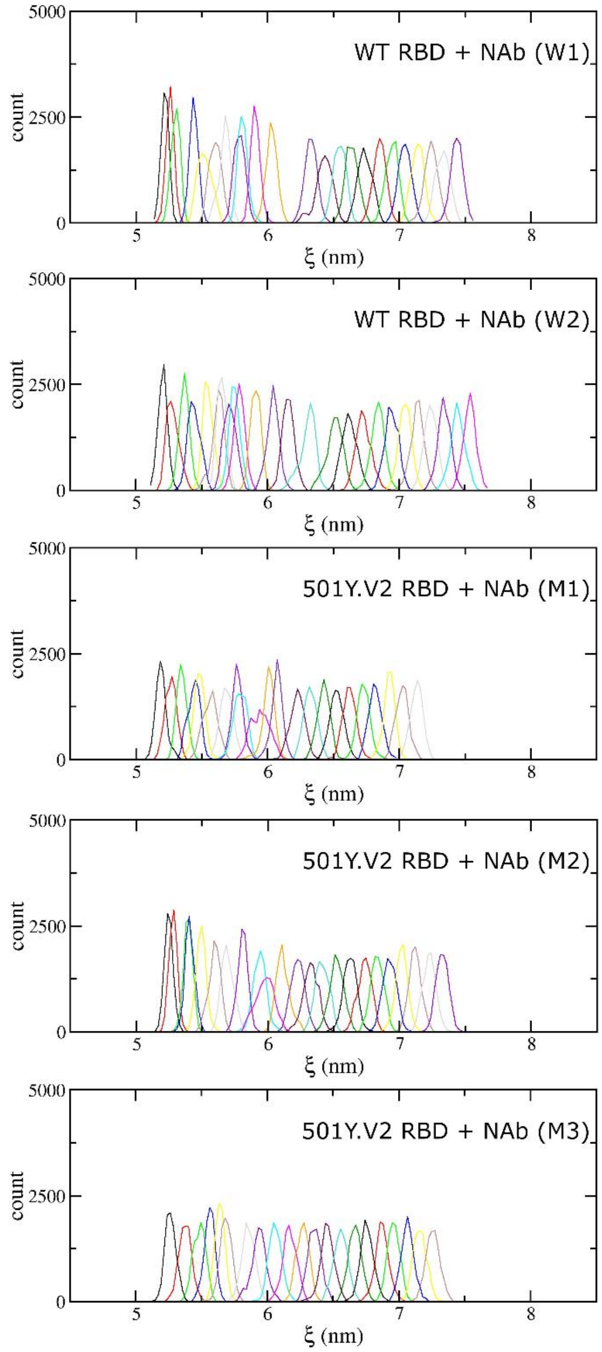

Figure S18. The histograms of US simulations over the unbinding process of NAb out of the WT/501Y.V2 RBD + NAb complexes.

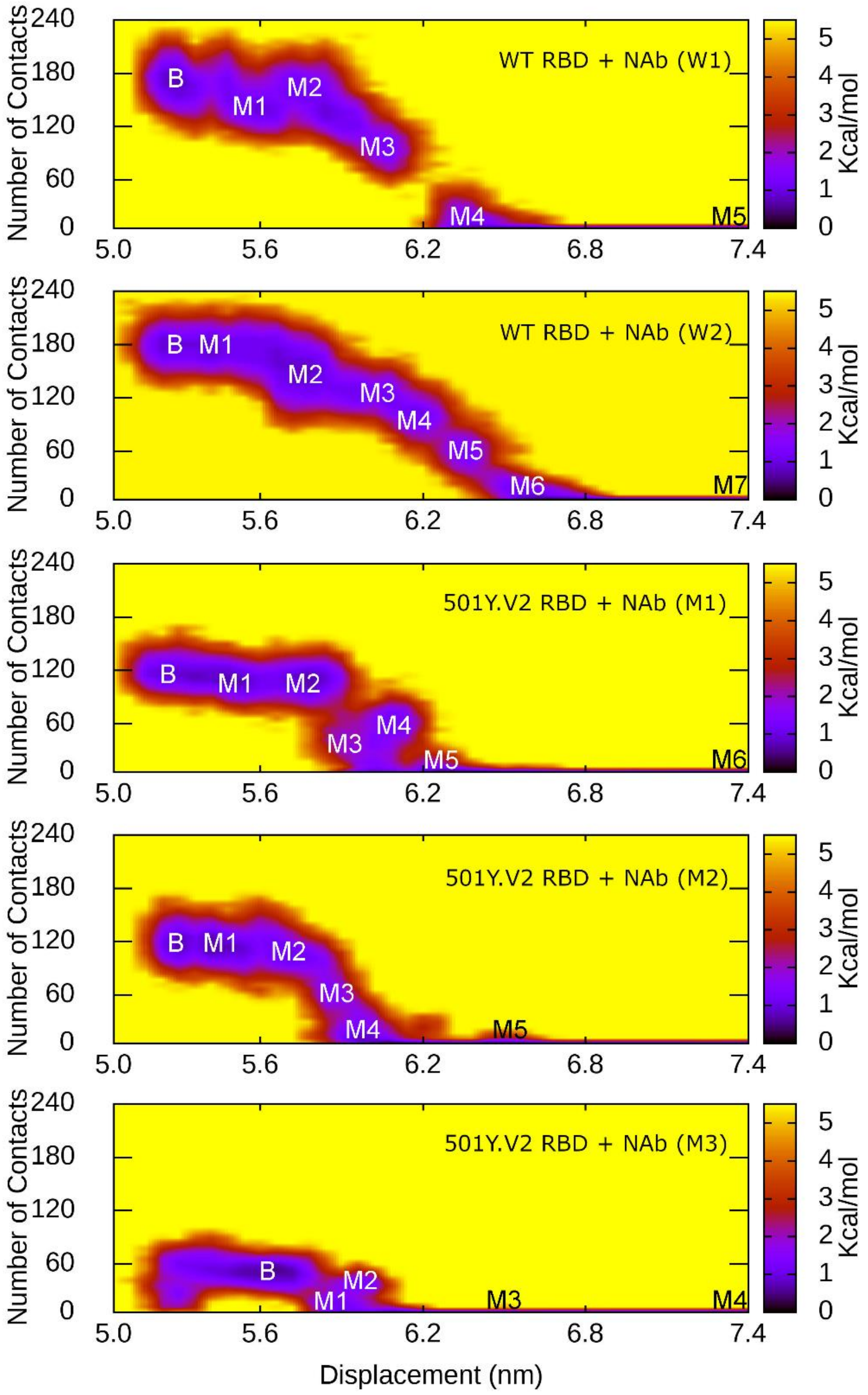

Figure S19. The collective-variable FEL revealed the unbinding pathways of NAb from the WT/501Y.V2 RBD + NAb complexes. B corresponds to the *bound* state. Mx respond to the dissociated state, in which x is range from 4 to 7.
